# An Atypical Presentation of Rocky Mountain Spotted Fever Presenting as Progressive Vision Loss: A Case Report

**DOI:** 10.7759/cureus.83782

**Published:** 2025-05-09

**Authors:** Hussein Abourahma, Sayf Adas, Tara Salimi, Cheryl N Gonzalez Figueroa, Erik Perez, Naaz Fatteh, Tye Barber

**Affiliations:** 1 Medical Student, Dr. Kiran C. Patel College of Osteopathic Medicine, Nova Southeastern University, Fort Lauderdale, USA; 2 Family Medicine, Broward Health Medical Center, Fort Lauderdale, USA; 3 Infectious Disease and Internal Medicine, Broward Health Medical Center, Fort Lauderdale, USA

**Keywords:** hiv, immunocompromised, ocular involvement, progressive vision loss, rocky mountain spotted fever

## Abstract

This case report describes a 49-year-old immunocompromised male with human immunodeficiency virus (HIV) who presented with progressive bilateral vision loss. He initially noticed blurriness and visual distortion in the right eye several months prior, which gradually progressed to involve the left eye with worsening peripheral vision and intermittent diplopia. Despite several evaluations and empiric treatment, his symptoms continued to worsen. Prior imaging and lumbar puncture (LP) were inconclusive. After extensive evaluation by ophthalmology, neurology, and infectious disease, the differential diagnosis was expanded to include infrequent causes, including infectious processes. A panel of diagnostic tests ultimately revealed Rocky Mountain Spotted Fever (RMSF). While it is typically a curable disease, RMSF can be a potentially lethal disease caused by the bacteria *Rickettsia rickettsii*. Human-to-human transmission is not known to occur. The patient denied tick bites or exposure risk; however, an unknown tick exposure could have occurred. At the time of diagnosis, the patient was significantly immunocompromised. Given this immunocompromised state, it is possible that the patient was more susceptible to infection transmission. Despite the absence of the classic triad of fever, rash, and headache, serological testing ultimately led to the diagnosis of RMSF, demonstrating the need for a broad differential and a high index of suspicion for this potentially life-threatening illness, especially in an immunocompromised patient.

## Introduction

Rocky Mountain spotted fever (RMSF) is a tick-borne illness caused by *Rickettsia rickettsii*. Despite its name, RMSF is most prevalent in the southeastern and south-central United States (U.S.). In the U.S., transmission is often more likely in the summer and caused by the American dog tick (*Dermacentor variabilis*), the Rocky Mountain wood tick (*Dermacentor andersoni*), or the brown dog tick (*Rhipicephalus sanguineus*) [1,2]. Rickettsia infects vascular endothelial cells, leading to systemic inflammation, increased vascular permeability, and multi-organ dysfunction. This can manifest as fever, myalgias, neurological symptoms, rash, and cardiovascular instability, contributing to its high mortality [3].

Although the classical diagnostic triad of fever, headache, and rash is well-known, studies show that many patients - especially early in the disease or those who are immunocompromised - may not present with all three symptoms, making early recognition challenging [4]. Early consideration is critical in RMSF because if treatment is delayed past the first five days of the illness, the severity of the disease and the probability of death are increased. Without prompt antibiotic treatment, mortality rates can be as high as 20% to 30% [3]. However, laboratory findings are often normal early in the disease, so the decision to treat is based on clinical suspicion [5]. Thus, treatment with doxycycline for seven to ten days should not be delayed while waiting for confirmatory laboratory testing in a patient with a suspected rickettsial infection.

This case report discusses the clinical course of a 49-year-old immunocompromised male who presented with bilateral progressive vision loss, which was ultimately diagnosed as RMSF. RMSF can be difficult to diagnose due to its nonspecific initial presentation, as seen in this patient; therefore, a broad differential is crucial. Early recognition and prompt antibiotic treatment are essential to prevent severe complications and improve outcomes.

## Case presentation

A 49-year-old male with a past medical history of HIV, chronic medical conditions including hypertension and anxiety, and a history of optic disc edema presented with progressive bilateral vision loss. He first noticed decreased vision in his right eye several months earlier, followed by similar symptoms in the left eye, which gradually worsened. He denied any trauma, pain with extraocular movements, headaches, or weakness but did endorse occasional diplopia. He had strabismus surgery in childhood and does not use corrective lenses. The patient was previously admitted a few months prior with a similar complaint, where testing, including magnetic resonance imaging (MRI), computed tomography (CT), and lumbar puncture (LP), was unremarkable. He was discharged with a six-week steroid taper and outpatient neurology and ophthalmology follow-up. The patient did not keep the follow-up appointments. Recently, the patient tested positive for HIV. On admission, the patient complained of a sore throat for three days but denied any recent travel, sick contacts, rash, fevers, nausea, or vomiting. Other than mild transaminitis and mild tachycardia, labs and vitals were initially normal.

Upon admission for further evaluation, ophthalmology, neurology, and infectious disease were all consulted. Testing showed he was significantly immunocompromised. MRI of the brain and orbit with/without contrast showed chronic sinusitis but was otherwise unremarkable. Ophthalmology noted optic disc pallor on exam, and LP results were not consistent with meningitis.

The patient was given empiric intravenous (IV) ganciclovir due to a low CD4 count and concern for cytomegalovirus (CMV) retinitis. Additional infectious workups included CMV polymerase chain reaction (PCR), histoplasmosis, cryptococcus, Lyme, rickettsia, and toxoplasmosis. Although RMSF was not initially high on the differential due to the low incidence in this geographic region, it was included in the broader panel out of caution, given the patient’s immunocompromised state and progressive symptoms. Rickettsial antibody testing returned positive for RMSF. Although RMSF typically presents with fever, headache, and rash, it can have ocular manifestations, including vasculitis. The patient was initiated on doxycycline 100 mg twice daily with reported improvement of his symptoms.

## Discussion

RMSF is a potentially fatal tick-borne illness caused by *Rickettsia rickettsii*, which primarily targets vascular endothelial cells and can lead to multisystemic small vessel vasculitis that affects the lungs, heart, skin, central nervous system, liver, and kidneys [6]. In this case, we discuss an atypical presentation of RMSF where an immunocompromised, HIV-positive, 49-year-old male presented with progressive bilateral vision loss as the chief complaint. Recent studies show that ocular involvement - especially retinitis, retinal vasculitis, and neuroretinitis - is more common than previously thought in Rickettsial infections [7]. In this case, the absence of the classic symptoms delayed the initial suspicion of RMSF, which ultimately led to a broader infectious disease workup that confirmed the diagnosis through serological testing.

In the United States, RMSF is relatively rare, with approximately 5,000-6,000 cases reported annually. The highest incidence is seen in North Carolina, Oklahoma, Arkansas, Tennessee, and Missouri, which collectively account for over 60% of reported cases. However, the severity of untreated RMSF and its potential for atypical presentations - particularly in immunocompromised patients - underscores the need to consider it even in low-incidence settings [8].

Immunocompromised patients, particularly those with HIV, may have altered disease presentations due to their impaired immune response. As seen in this patient, significant immunosuppression raised concern for opportunistic infections such as CMV retinitis, prompting empiric treatment with IV ganciclovir. However, CMV testing was negative, which led to the decision to broaden the differential diagnosis and infectious workup, ultimately leading to RMSF. Early empiric antibiotic therapy is essential to prevent severe complications or a fatal outcome, but the disease often presents as a dilemma for clinicians because of its nonspecific presentation [9]. The standard treatment of doxycycline remains effective regardless of the patient's age or immune status.

This case emphasizes the importance of maintaining a broad differential diagnosis when evaluating vision loss, especially in immunocompromised patients, where the etiology is unclear. A full workup, including autoimmune, vascular, and infectious, is essential. The patient's presentation here, while atypical, underscores the need for clinicians to recognize and initiate early treatment in suspected cases even in the absence of classical symptoms. Future research should focus on the relationship between RMSF and ocular manifestations, particularly in immunocompromised individuals, to improve early recognition and management strategies.

## Conclusions

This case underscores the importance of considering atypical presentations of infectious diseases, including RMSF, particularly in immunocompromised individuals. A thorough history, physical examination, and appropriate laboratory testing are crucial for accurate diagnosis and timely treatment. The successful identification of RMSF in this patient, despite the absence of typical symptoms, highlights the value of maintaining a broad differential and pursuing further investigations when the clinical picture is unclear. This case also emphasizes the need for clinicians to be aware of the varied manifestations of RMSF, including ocular involvement, to ensure prompt and appropriate management and improve patient outcomes.
